# Heart failure with preserved ejection fraction based on aging and comorbidities

**DOI:** 10.1186/s12967-021-02935-x

**Published:** 2021-07-06

**Authors:** Ying Lin, Shihui Fu, Yao Yao, Yulong Li, Yali Zhao, Leiming Luo

**Affiliations:** 1 Department of Cardiology, Hainan Hospital of Chinese People’s Liberation Army General Hospital, Sanya, 572013 China; 2 Department of Geriatric Cardiology, Chinese People’s Liberation Army General Hospital, Beijing, 100853 China; 3 Centre for the Study of Ageing and Human Development and Geriatrics Division, Medical School of Duke University, Durham, NC 27708 USA; 4 Centre for Healthy Ageing and Development Studies, National School of Development, Peking University, Beijing, 100871 China; 5 Central Laboratory, Hainan Hospital of Chinese People’s Liberation Army General Hospital, Sanya, 572013 China

**Keywords:** Aging, Comorbidities, Diagnosis, Heart failure with preserved ejection fraction, Treatment

## Abstract

Heart failure (HF) with preserved ejection fraction (HFpEF) is a leading cause of hospitalizations and mortality when diagnosed at the age of ≥ 65 years. HFpEF represents multifactorial and multisystemic syndrome and has different pathophysiology and phenotypes. Its diagnosis is difficult to be established based on left ventricular ejection fraction and may benefit from individually tailored approaches, underlying age-related changes and frequent comorbidities. Compared with the rapid development in the treatment of heart failure with reduced ejection fraction, HFpEF presents a great challenge and needs to be addressed considering the failure of HF drugs to improve its outcomes. Further extensive studies on the relationships between HFpEF, aging, and comorbidities in carefully phenotyped HFpEF subgroups may help understand the biology, diagnosis, and treatment of HFpEF. The current review summarized the diagnostic and therapeutic development of HFpEF based on the complex relationships between aging, comorbidities, and HFpEF.

## Introduction

Heart failure (HF) is divided into three forms based on left ventricular (LV) ejection fraction (LVEF): heart failure with preserved ejection fraction (HFpEF, LVEF ≥ 50%), heart failure with reduced ejection fraction (HFrEF, LVEF < 40%), and heart failure with mid-range ejection fraction (HFmEF, LVEF ≥ 40 and < 50%) [1]. HFpEF has a global prevalence of 2% and will increase by 50% by 2035 in aging populations [2]. Patients suffering from HFpEF are older, mostly female and obese, and exhibit a lower prevalence of coronary artery disease (CAD) than patients with HFrEF [3, 4]. Nearly all patients with HF have preserved EF in the elderly ≥ 90 years [5, 6]. Atrial fibrillation (AF) increases subsequent HF risk five-fold during the following ten years [7]. Patients with AF and underlying HFpEF have reduced exercise tolerance and worsened ventricular function than those with AF alone [8–11]. HFpEF is a leading cause of hospitalizations and mortality when diagnosed at the age of ≥ 65 years. Patients with HFpEF have higher morbidity, mortality, and rehospitalization as those with HFrEF, and life quality in patients with HFpEF is worse than in those with HFrEF [12]. The complex interaction between aging and comorbidities makes HFpEF a significant burden to public health. The current review summarized the diagnostic and therapeutic development of HFpEF based on the complex relationships between aging, comorbidities, and HFpEF.

## Pathophysiology

HFpEF is a systemic syndrome involving multiple organs [13]. Diastolic factors affecting HFpEF are the pulmonary vein (preload), vascular resistance (afterload), and contractility relaxation (cardiac). Contractility is disturbed by atrial function, ventricular dyssynchrony, and atrioventricular maladjustment [14]. Relaxation of myocardial tissue is achieved through energy-dependence myofilament dissociation and passive relaxation of noncardiomyocyte matrix in the cardiac chambers and pericardium [15–17].

HFpEF is triggered by the cumulative expression of various risk factors and comorbidities, including age, sex (female), physical inactivity, obesity, AF, CAD, diabetes, dyslipidemia, hypertension, metabolic syndrome, chronic kidney disease, anemia, chronic obstructive pulmonary disease and sleep-disordered breathing [18]. However, there are no specific diseases demonstrated to be etiologic of HFpEF. HFpEF is a systemic inflammatory or metabolic disorder [19]. First, HFpEF is associated with endothelial inflammation, leading to coronary microvascular dysfunction [20, 21]. Endothelial dysfunction is a significant factor linking cardiac and extracardiac effectors [22]. Second, the changed composition and structure of both cardiomyocytes and noncardiomyocytes can increase diastolic stiffness and promote HFpEF development [23–25]. Third, both obesity and diabetes are accompanied by increased epicardial adipose tissue volume, which transduces the effects of these diseases on cardiac function and structure [26]. HFpEF is a microcirculation defect following the obesity and diabetes. Both obesity and diabetes lead to an inflammatory and fibrotic atrial and ventricular myopathy, the two major elements of HFpEF [27]. Obesity and diabetes increase the risk of exercise intolerance and promote rapid progression of HFpEF due to multimorbidity, impaired chronotropic reserve, left ventricular hypertrophy, and activation of inflammatory, pro-oxidative, vasoconstrictor, and profibrotic pathways [28].

Although LVEF is not reduced, increased LV-filling pressure results in exertional dyspnea and exercise intolerance. If dysfunctional epicardial adipose tissue is adjacent to LV, it impairs LV distensibility and promotes HFpEF development. However, if it is adjacent to left atrium (LA), atrial myopathy is caused by electro-anatomical fragmentation and structural remodeling of LA [29]. AF may be the first indicator of an inflammatory or metabolic LA myopathy causing HFpEF [30]. Patients with HFpEF and AF, especially patients at increased risk of adverse outcomes, have increased epicardial adipose tissue volume [31]. AF reflects the development of myocardial inflammation, fibrosis, and hypertrophy in parallel with atrial and ventricular myopathy that results in HFpEF. Myocardial inflammation, fibrosis, and hypertrophy are identified in LA and LV of both patients with AF and those with HFpEF. Atrium and ventricle may be adversely affected by inflammation, and myocardial fibrosis and hypertrophy may contribute to exercise intolerance [32]. Cardiometabolic abnormalities, such as abnormal mitochondrial function, changed substrate utilization, and intracellular calcium overload, are also considered pathophysiological mechanisms in HFpEF [33].

Aging affects pathophysiological process of HFpEF. Structural and functional changes related to aging are generally believed to be significant risk factors of HFpEF [34]. Aging results in changed body composition, missed muscle mass, and increased sarcopenic adiposity [35]. Both aging and HFpEF are associated with changed epicardial adipose and its secretory adipocytokines. The elderly with HFpEF have 5.5 noncardiac comorbidities on average [36]. Aging, frailty, and comorbidities have cumulative and synergistic effects on cardiac function and outcomes [37]. Aging promotes coronary microvascular endothelial abnormalities and myocardial remodeling and dysfunction in HFpEF [38–40].

A key obstacle for exploring new pathophysiological mechanisms and testing new pharmaceutical substances is the availability of suitable animal models for HFpEF, which realistically reflect the research and clinical picture. A variety of animal models are developed with the signs of HFpEF ranging from murine models to a pig model. Most of these animal models develop HFpEF triggered by a single factor like hypertension (Dahl salt‐sensitive rat, aldosterone‐infused uninephrectomized mouse, and transverse aortic constriction‐induced pressure overload in mouse), obesity/diabetes (db/db mouse), and aging (senescence‐accelerated mouse). Schiattarella and colleagues recently formulated a ‘two‐hit’ hypothesis, inducing HFpEF in mice by metabolic stress (feeding of a high fat diet) and mechanical stress (hypertension induced by blocking eNOS activity) as second stressor. However, another animal model, developing HFpEF owing to diabetes and hypertension, is the ZSF1 (Zucker fatty and spontaneously hypertensive) rat. This model was developed by crossing rat strains with two separate leptin receptor mutations (fa and facp), the lean female ZDF rat (+ /fa) and the lean male SHHF rat (+ /facp). Offspring being homozygous for both mutations (fa:facp) are obese and develop insulin resistance, hyperglycaemia, and mild hypertension (ZSF1‐obese). The ZSF1‐obese animals developed HFpEF signs, exercise intolerance, reduced skeletal muscle contractility and endothelial dyfunction. ZSF1 rat may serve as a suitable animal model to study pathophysiological mechanisms and pharmaceutical strategies for HFpEF.

## Diagnosis

HFpEF presents a significant challenge in the diagnostic process, lacking a useful and objective one-method-fit-all approach [41]. Diagnosing HF in the elderly poses specific challenges to specialized physicians as false-positive and false-negative diagnosis are common in clinical practice [42]. First, exercise intolerance often happens in the elderly or obese population and represents pathophysiologic changes associated with aging or noncardiac etiologies [43, 44]. Second, HFpEF may be difficult to be diagnosed in the elderly because of existing comorbidities, which mimic HFpEF clinical manifestation and further complicate its diagnosis [45]. Third, the elderly with HFpEF have no classic HF manifestations. Circulating B-type natriuretic peptide (BNP) levels may not represent LV-filling pressure in patients with HFpEF. Patients with increased LV-filling pressures related to HFpEF commonly have no elevated BNP levels, possibly because distensibility is impaired by myocardial fibrosis or due to coexistent obesity [46]. Patients with HFpEF often have BNP levels below typical diagnostic thresholds. Most studies have suggested that around 30% of HFpEF patients have a BNP < 100 pg/ml, challenging the common practice of using BNP levels to determine HF diagnosis [47]. Fourth, HFpEF cannot be diagnosed based on diastolic dysfunction by itself due to the lack of a universally agreed definition. Finally, the limited ability of echocardiographic variables in identifying diastolic dysfunction further challenges its diagnosis in clinical practice [48].

Cardiac magnetic resonance imaging provides structural evidence of HFpEF, such as increased epicardial adipose tissue volume and myocardial fibrosis. Cardiac catheterization is the best method to confirm increased LV-filling pressure, but being an invasive method, its application in the elderly is limited. Electrocardiography showing AF may be an available and sensitive marker of HFpEF in the elderly. Diastolic stress testing is of essential significance for diagnosing HFpEF [49–53]. HFpEF with diastolic dysfunction results in increased LV end-diastolic pressure (LVEDP) to generate sufficient cardiac output for the peripheral tissue's needs. Elevated LVEDP or reduced end-organ perfusion is the most significant indicator for HFpEF diagnosis [54, 55]. Genetic analysis, imaging, and biopsy have been recommended to determine the etiologies of HFpEF.

**Fig. 1:**
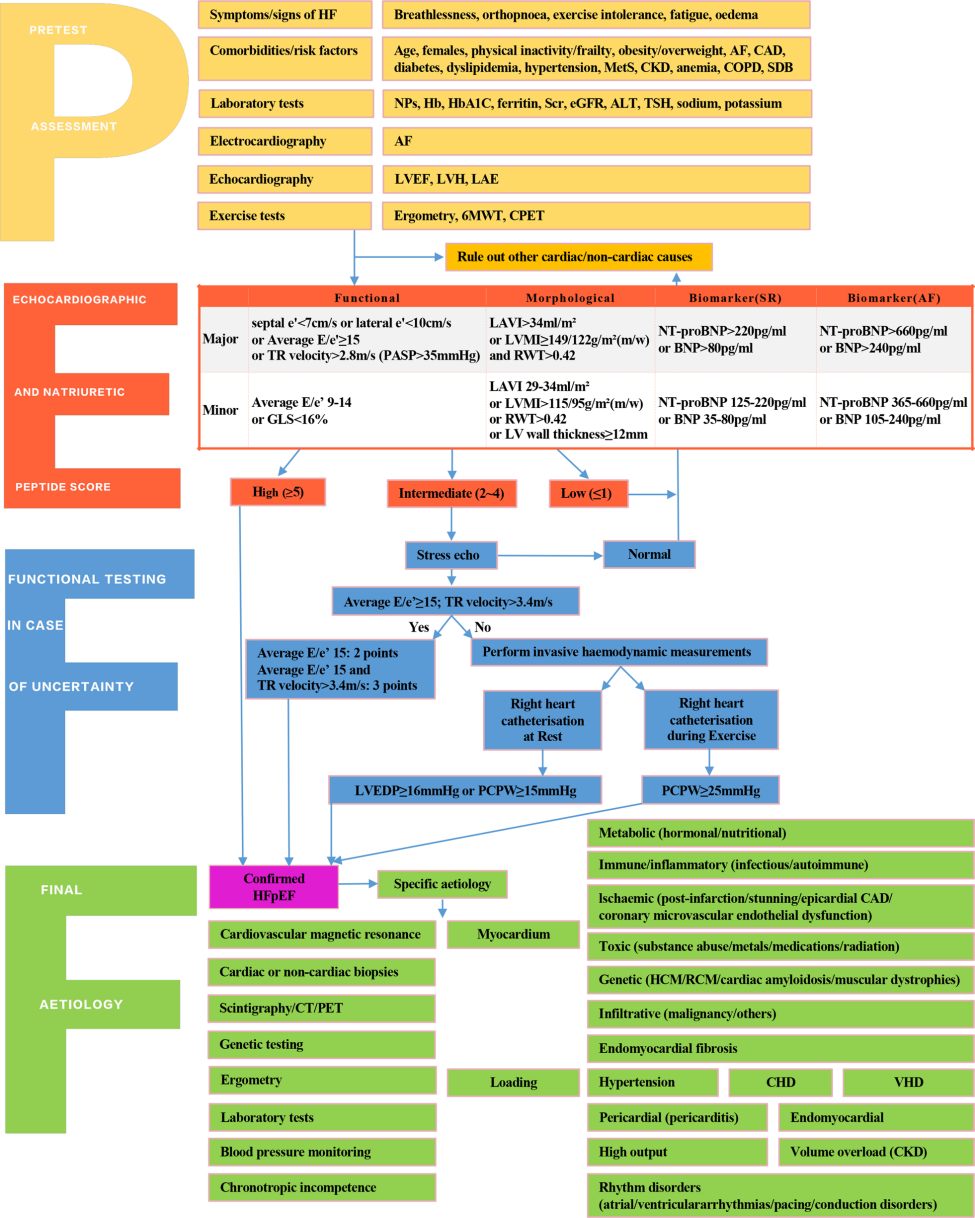
Heart Failure Association Pretest assessment, Echocardiography, and natriuretic peptide, Functional testing, Final etiology (HFA-PEFF): a diagnostic procedure for heart failure with preserved ejection fraction HFpEF. *HF* heart failure, *AF* atrial fibrillation, *CAD* coronary artery disease, *MetS* metabolic syndrome, *CKD* chronic kidney disease, *COPD* chronic obstructive pulmonary disease, *SDB* sleep-disordered breathing, *NPs* natriuretic peptides, *Hb* hemoglobin, *HbA1C* hemoglobin A1C, *Scr* serum creatinine, *eGFR* estimated glomerular filtration rate, *ALT* alanine aminotransferase, *TSH* thyroid stimulating hormone, *LVEF* left ventricular ejection fraction, *LVH* left ventricular hypertrophy, *LAE* left atrial enlargement, *6MWT* 6 min walk test, *CPET* cardiopulmonary exercise testing, *TR* tricuspid regurgitation, *PASP* pulmonary artery systolic pressure, *GLS* global longitudinal strain, *LAVI* left atrial volume index, *LVMI* left ventricular mass index, *RWT* relative wall thickness, *LV* left ventricular, *SR* sinus rhythm, *NT-proBNP* N-terminal pro-B-type natriuretic peptide, *BNP* B-type natriuretic peptide, *LVEDP* left ventricular end-diastolic pressure, *PCWP* pulmonary capillary wedge pressure, *CT* computed tomography, *PET* positron emission tomography, *HCM* hypertrophic cardiomyopathy, *RCM* restrictive cardiomyopathy, *CHD* congenital heart disease, *VHD* valvular heart disease

HFpEF may be represented by different pathophysiological phenotypes, which require differential identification and management [56]. Diagnostic algorithms with a series of measures include clinical, laboratory, and instrumental characteristics; sophisticated imaging modalities; and invasive hemodynamic measurements. As shown in Fig. 1, Heart Failure Association Pretest assessment, Echocardiography, and natriuretic peptide, Functional testing, Final etiology (HFA-PEFF) has been suggested as a diagnostic procedure for HFpEF [57]. Additionally, a weighted score based on obesity, AF, age > 60 years, treatment with ≥ 2 antihypertensives, echocardiographic E/e' ratio > 9, and echocardiographic pulmonary artery systolic pressure > 35 mmHg was used to create a composite score (H_2_FPEF score) ranging from 0 to 9. The odds of HFpEF doubled for each 1-unit score increase. H_2_FPEF score relies on simple clinical characteristics, whereas cardiac catheterization is necessary in HFA-PEFF. H_2_FPEF score enables the discrimination of HFpEF from noncardiac causes of dyspnea and determine further diagnostic testing in the evaluation of patients with unexplained dyspnea.

## Prevention

The prevention of HF is hard and controlling risk factors may be feasible. Lifestyle modifications, such as dietary control, nutrient management, physical activity, weight loss, and cardiorespiratory fitness, have beneficial effects on the prevention of HFpEF [58]. Treating obesity or diabetes can affect the volume or function of epicardial adipose tissue. Both caloric restriction and physical activity are effective methods to improve cardiac outcomes in patients with HFpEF. Caloric restriction and weight loss significantly improve exercise tolerance and life quality in the elderly with HFpEF and obesity [59–61]. Weight loss reduces the risk of HFpEF, lowers elevated diastolic filling pressure, and alleviates epicardial adipose inflammation [62–64]. Exercise protocols mainly include aerobic exercise, such as walking or cycling, in the elderly. Exercise training at home can be achieved by remote monitoring, individualization programme, and fall prevention. Exercise training improves physical function, shows clear security, and reduces HFpEF rehospitalization in the elderly [65]. Moderate and regular physical activity is recommended in patients with HFpEF by the American College of Cardiology/American Heart Association (ACC/AHA).

Addressing the risk factors and comorbidities is a significant way of preventing the development of HFpEF [66, 67]. First, hypertension can obviously increase prevalence, rehospitalization and mortality of patients with HFpEF; thus, treating hypertension may be the most effective prevention method for HFpEF [68, 69]. Second, CAD deteriorates ventricular function and outcomes and increases the occurrence of HFpEF, and patients with CAD patients should receive systemic treatment, such as coronary revascularization [70, 71]. Third, because of increased longevity, AF has increasing prevalence and coexists with HFpEF [72]. AF is closely related to abnormal atrial and ventricle function, neurohumoral activation, and exercise intolerance [73]. Tachycardia is also deleterious by shortening diastole time and impairing diastolic filling. Rate or rhythm control of AF may prevent the development of an underlying HFpEF. Rate control and permanent anticoagulation are recommended in patients with AF [74, 75]. Finally, anemia is related to elevated prevalence, hospitalization and mortality of HFpEF [76, 77]. Enhancing mitochondrial energy by iron supplementation prevents the development of HFpEF, and iron supplementation rather than erythropoietin is recommended by the ACC/AHA [78].

## Treatment

HFpEF, being one of the most challenging diseases to treat, does not respond to a one-method-fit-all approach; hence, several therapeutic methods are shown in Table 1 [79–82]. Angiotensin-converting enzyme inhibitors and angiotensin receptor blockers (ARBs) can alleviate the inflammation of adipose and attenuate myocardial fibrosis and remodeling [83]. They improve clinical symptoms and exercise tolerance rather than morbidity or mortality in patients with HFpEF [84]. Moreover, they do not cause further improved exercise tolerance or cardiac function after optimal diuretic treatment [85, 86]. Aldosterone mediates myocardial fibrosis, contributing to myocardial stiffness [87]. Mineralocorticoid receptor antagonists fail to improve clinical symptoms, exercise tolerance, and cardiac outcomes in patients with HFpEF [88].

**Table 1:** Trials of exercise, medications and devices in patients with HFpEF

Types	Interventions	Inclusion	Trials	Endpoints	Results
Exercise	Exercise training	NYHA II-III, EF ≥ 50%, tissue Doppler-derived E/e' ratio	Ex-DHF	Exercise capacity, QOL	Positive
ACEI/ARB	Candesartan	Aged ≥ 18 years, NYHA II-IV, EF > 40%	CHARM-Preserved	CV death, HF hospitalization	Neutral
	Perindopril	Aged ≥ 70 years, clinical diagnosis of chronic HF, EF ≥ 40%, hospitalised for a cardiac problem, able to walk without the aid of another person	PEP-CHF	CV death, HF hospitalization	Neutral
ARNI	Sacubitril/valsartan	Aged ≥ 40 years, EF ≥ 45%, HF signs or symptoms, NT-proBNP ≥ 400 pg/mL, eGFR ≥ 30 mL/min/1.73m^2^, potassium ≤ 5.2 mmol/L	PARAMOUNT	NT-proBNP	Positive
		Aged ≥ 45 years, EF > 40%, LAE or LVH on echocardiography, NYHA II–IV, NT-proBNP > 220 pg/mL for patients with no AF or > 600 pg/mL for those with AF	PARALLAX	NT-proBNP	Positive
sGC stimulator and activator	Vericiguat	Aged ≥ 45 years, EF ≥ 45%, NYHA II–III, HF decompensation, NT-proBNP ≥ 300 or BNP ≥ 100 pg/mL in sinus rhythm, or NT-proBNP ≥ 600 or BNP ≥ 200 pg/mL in AF, LVH (intraventricular septal or posterior wall thickness ≥ 1.1 cm, and/or LVMI ≥ 115 g/m^2^ in male and ≥ 95 g/m^2^ in female), or LAE (LAV index ≥ 29 mL/m^2^, or LAV > 58 mL in male and > 52 mL in female patients, or LA area > 20 cm^2^, or LA diameter > 40 mm in male and > 38 mm in female patients)	VITALITY	QOL	Positive
		NYHA II-IV, EF ≥ 45%, BNP ≥ 100 pg/mL or NT-proBNP ≥ 300 pg/mL(or BNP ≥ 200 pg/mL or NTproBNP ≥ 600 pg/mL in AF), LAE determined by echocardiography	SOCRATES-PRESERVED	QOL	Neutral
	Riociguat	EF ≤ 35%, NYHA Class III-IV	DYNAMIC	CO	Positive
SGLT-2 inhibitor	Empaglifozin	NYHA II-IV, EF > 40%, NT-proBNP > 300 pg/mL in patients without AF and > 900 pg/mL in AF, structural changes in the heart (left atrial size or LVM) on echocardiography, HF hospitalization	EMPEROR-PRESERVED	CV death, HF hospitalization	Positive
		EF > 40%, NYHA II-IV, 6MWD of ≥ 100 m and ≤ 350 m	EMPERIAL-PRESERVED	6MWD	Positive
	Sotaglifozin	Type 2 diabetes mellitus, HF hospitalization	SOLOIST-WHF	CV death, HF hospitalization	Positive
Nitrate	Oral nitrate	Mean PAP ≥ 35 mmHg and baseline PCWP ≥ 20 mmHg, NYHA II-III, EF ≥ 40%	PH-HFPEF	PAP at exercise	Positive
MRA	Spironolactone	Aged ≥ 50 years, EF ≥ 45%, potassium < 5.0 mmol/L, HF hospitalization, BNP ≥ 100 pg/ml, NT-proBNP ≥ 360 pg/ml	TOPCAT	HF hospitalization	Neutral
		Aged ≥ 50 years, NYHA II-III, EF 50%, diastolic dysfunction	Aldo-DHF	Neurohumoral activation, LVH	Positive
PDE-5 inhibitor	Sildenafl	Outpatients with HFpEF	RELAX	PAP, CO	Positive
Pirfenidone	Pirfenidone	Aged ≥ 40 years, EF ≥ 45%, symptoms and signs of HF, BNP ≥ 100 pg/ml or NT-proBNP ≥ 300 pg/ml(patients in AF are required to have BNP ≥ 300 pg/ml or NT-proBNP ≥ 900 pg/ml)	PIROUETTE	ECV	Positive
Cardiolipin peroxidase inhibitor	Elamipretide	Aged 40–80 years, EF ≤ 40%, no hospitalization related to HF, at least 3 dysfunctional but viable segments (hyperenhancement ≤ 25%) by cardiac MRI examination	PROGRESS-HF	NT-pro-BNP	Positive
Beta3-adrenoreceptor selective agonist	Mirabegron	LVH (increased LVMI or LVWT ≥ 13 mm in at least one wall segment), in the absence of genetic hypertrophic cardiomyopathy and significant valvular disease	BETA3-LVH	LVMI, E/e′	Positive
Device therapy	CardioMEMS	NYHA II-IV regardless of EF with and elevated natriuretic peptides	GUIDE-HF	All-cause death, HF hospitalization	Positive
		HF ≥ 3 months, NYHA class III	CHAMPION	HF hospitalization	Positive
	IASD	EF ≥ 40% and NYHA III-IV HF, PCWP ≥ 15 mmHg at rest or ≥ 25 mmHg during supine bike exercise	REDUCE LAP-HF	CV death, HF hospitalization	Positive
	ASV	HFpEF or HFrEF, AHI ≥ 15 events per hour	CAT HF	CV death, HF hospitalization, 6MWD	Positive

*HFpEF* heart failure with preserved ejection fraction, *NYHA* New York Heart Assocation, *AF* atrial fibrillation, *QOL* quality of life, *6MWD* 6-min walk distance, *ACEI* angiotensin-converting enzyme inhibitor, *ARB* angiotensin receptor blocker, *ARNI* angiotensin receptor-neprilysin inhibitor, *eGFR* estimated glomerular filtration rate, *CV* cardiovascular, *BNP* B-type natriuretic peptide, *NT-proBNP* N-terminal pro-B-type natriuretic peptide, *sGC* soluble guanylyl cyclase, *LAE* left atrial enlargement, *CO* cardiac output, *PCWP* pulmonary capillary wedge pressure, *SGLT-2* sodium glucose cotransporter-2, *HF* heart failure, *PAP* pulmonary artery pressure, *LVH* left ventricular hypertrophy, *PDE-5* phosphodiesterase-5, *MRA* mineralocorticoid receptor antagonist, *ECV* extracellular volume fraction, *MRI* magnetic resonance imaging, *E/e′* mitral early diastolic velocity/mitral annular velocity, *LVMI* left ventricular mass index, *IASD* interatrial shunt device, *ASV* adaptive servo-ventilation, *HFrEF* heart failure with reduced ejection fraction, *AHI* apnea–hypopnea index

Tachycardia predicts poor outcomes in patients with HFpEF [89]. Beta-blockers have no prognostic effect in patients with HFpEF but may have beneficial roles in some subgroup analyses [90]. Conventional beta-blockers mainly target beta1- and beta2-adrenoreceptors, which can mediate catecholamine effects. Beta3-adrenoreceptor prevents neurohormonal stimulation and myocardial hypertrophy [91]. Stimulating Beta3-adrenoreceptor with selective agonist mirabegron may be studied as a treatment method in HFpEF. Although ivabradine reduces exercise-induced tachycardia and improves chronotropic incompetence, it cannot prolong diastole time to restore diastolic function in HFpEF [92]. HFpEF has overfilled LV but not impaired LV filling, thus invalidating traditional rationale for slowing heart rate [93, 94]. Cardiac glycosides, such as digoxin, cannot improve cardiac mortality but treat the tachyarrhythmia in HFpEF [95]. However, atrioventricular node blocking drugs, such as digoxin, can exert lethal proarrhythmic effects independent of slowing heart rate [96, 97]. A pacemaker is indispensable to treat conduction system disease in patients with HFpEF, particularly in those with AF [98, 99]. Rhythm control, such as cardioversion or catheter ablation, is considered when AF is associated with clinical symptoms of patients with HFpEF. Intensive application of membrane-active anti-arrhythmic drugs poses a risk to the development of arrhythmia and HFpEF [100, 101]. Catheter ablation restores sinus rhythm and improves LV function but is not effective in patients with myocardial fibrosis [102–106].

Statins can decrease epicardial adipose tissue volume and thereby prevent systemic inflammation and myocardium fibrosis [107, 108]. Statins reduce new-onset and recurrent AF and further prevent AF-related thromboembolic events [109]. Meanwhile, the application of statins is followed by improved diastolic dysfunction and reduced HFpEF risk [110].

Natriuretic peptides activate guanylyl cyclase, resulting in cyclic guanosine monophosphate (cGMP) formation and preventing myocardial fibrosis due to vasodilation and diuresis [111]. Endogenous natriuretic peptides by neprilysin inhibition may produce an antiadipogenic effect on the epicardium [112]. The addition of neprilysin inhibition to ARBs [sacubitril/valsartan; angiotensin receptor-neprilysin inhibitor (ARNI)] ameliorates atrial and ventricular myopathy in patients with HFpEF [113]. Although sacubitril/valsartan increases plasma natriuretic peptides levels by inhibiting neprilysin, it failed to reduce cardiac mortality in the PARADIGM-HF trial [114]. However, subgroup analyses have demonstrated its efficacy in female patients and those with HFmEF [115]. There is evidence from a meta-analysis that sacubitril/valsartan in HFpEF probably reduces HFpEF hospitalization but probably has little or no effect on cardiovascular mortality and life quality. There is a need for improved approaches to patient stratification to identify the subgroup of patients with HFpEF who are most likely to benefit from sacubitril/valsartan, as well as for an improved understanding of biology, and for new therapeutic approaches of HFpEF.

Abnormal nitrogen monoxide-cGMP-protein kinase G (NO-cGMP-PKG) pathway may constitute a pathophysiological mechanism promoting myocardial fibrosis and diastolic dysfunction in HFpEF [116, 117]. Direct nitric oxide (NO) donators, including organic nitrates (isosorbide-nitrate), are not recommended in patients with HFpEF, considering their disadvantages of vasodilatation and hypotension [118]. They also fail to increase exercise tolerance and improve diastolic function [119]. Enhancing endothelial nitric oxide synthase activity by the transcription amplifier AVE3085 increases NO production and improves diastolic function [120]. However, this method is still pending clinical evaluation. Nitrosative stress is a major driver in HFpEF rather than the limited bioavailability of NO, with new strategies targeting nitrosative stress in the future [121].

Phosphodiesterase-5 inhibitors are applied to treat precapillary pulmonary arterial hypertension (PAH) and may be considered in combined precapillary and postcapillary PAH. Sildenafil fails to significantly lower pulmonary artery pressure (PAP) in HFpEF patients without precapillary PAH and those with postcapillary PAH [122, 123]. However, sildenafil has positive effects in HFpEF patients with precapillary PAH or severe combined precapillary and postcapillary PAH [124]. Soluble guanylyl cyclase activators, such as vericiguat and riociguat, are administered in patients with PAH. Vericiguat has recently been demonstrated to reduce cardiac mortality in patients with HFrEF [125]. Further studies will assess its effects on cardiac function and outcomes in patients with HFpEF.

Insulin has hypoglycemic, antinatriuretic, and adipogenic effects and causes adverse outcomes in patients with HFpEF [126, 127]. Metformin reduces proinflammatory adipokines and has anti-inflammatory roles [128, 129]. It reduces the risk of AF and improves diastolic dysfunction in HFpEF [130]. Metabolic abnormalities and systemic inflammation impair the expression of peroxisome proliferator-activated receptor (PPAR), but co-stimulation of PPAR and adiponectin reverses epicardial adipose tissue dysfunction [131, 132]. Pioglitazone and rosiglitazone suppress atrial and ventricular inflammation and fibrosis and reduce the risk of AF and HFpEF [133, 134]. Thiazolidinediones have been associated with an improved diastolic filling abnormality in patients with diabetes [135]. However, they promote sodium retention, thereby increasing cardiac volume [136]. Sodium retention may aggravate cardiac fibrosis and hypertrophy and increases the risk of HFpEF [137]. Sodium-glucose cotransporter-2 (SGLT2) inhibitors, such as dapagliflozin and empagliflozin, achieve significantly decreased primary composite endpoint of worsened HF or cardiac mortality in patients with HFrEF, which is independent of diabetes [138, 139]. SGLT2 inhibitors reduce the volume of epicardial adipose and cardiac events caused by HFpEF [140]. A number of randomized trials are underway to explore the efficacy of SGLT-2 in patients with HFpEF [141, 142]. However, because the patients in these studies did not demonstrate any HF-related manifestations or the degree of HF was low at baseline, any recommendation of SGLT2is for the treatment of HFpEF should be cautious.

Regulation of incretin system includes mimicking glucagon-like peptide 1 (GLP-1) roles and inhibiting GLP-1-degrading enzyme dipeptidyl peptidase-4 (DPP-IV) [143]. GLP-1 analogues, such as semaglutide and liraglutide, improve cardiac outcomes in patients with diabetes [144]. LIVE trial determined that although liraglutide did not affect LV function compared with placebo in stable HF patients with and without diabetes, treatment with liraglutide was associated with more serious cardiac adverse events [142]. FIGHT trial revealed that the use of liraglutide did not lead to greater post-hospitalization clinical stability [143]. The results of existing evidence do not support the use of liraglutide or semaglutide in HF with diabetes, and LIVE points out the potential harmful effect of liraglutide in this population. The safety of these powerful GLP-1 analogues in patients with diabetes and HF remains uncertain, and further studies are needed to assess their risks and benefits especially in patients with HFpEF.

As an anti-fibrotic drug, pirfenidone suppresses the development of ventricular fibrosis and diastolic dysfunction through targeting transforming growth factor β (TGF-β) signaling pathway in pressure-overload induced HF [145, 146]. Future studies would assess whether these effects account for patients with HFpEF [147, 148]. Lysyl oxidase-like 2 (Loxl2) promotes collagen's cross-linking and causes interstitial fibrosis [149]. Diastolic function may be improved by antibody-mediated inhibition of Loxl2 [150]. Inhibition of Loxl2 and new cross-linking strategies will be assessed in the future. Systemic inflammation is the main mediator in HFpEF, and cytokine inhibitors have been considered therapeutic options [151]. Although interleukin-1 (IL-1) blockade with anakinra cannot improve exercise tolerance, canakinumab, a monoclonal antibody targeting IL-1ß, decreases HF hospitalization and mortality [152].

Cardiolipin is a significant phospholipid in the inner mitochondrial membrane, and Szeto-Schiller (SS) peptide is an antioxidant peptide binding to cardiolipin [153]. Elamipretide (MTP-131, SS31) reduces LVEDP in patients with HFpEF and needs to be further assessed through clinical studies [154, 155]. Neladenoson bialanate, a partial adenosine A1 receptor agonist, may benefit both cardiac and skeletal muscles. It enhances SERCA2a activity and reverses ventricular remodeling through improving mitochondrial function but fails to significantly affect exercise tolerance in patients with HFpEF [156]. Levosimendan has positive inotropic and vasodilative effects through a combined effect on calcium sensitization and phosphodiesterase-3 inhibition. It improves inflammatory process and diastolic function in patients with HFrEF [157]. Meanwhile, inhaled iloprost causes an acute reduction of PAP in patients with HFpEF [158]. Further studies will assess levosimendan and prostacyclin analogs in patients with HFpEF. Fluid overload can aggravate clinical symptoms and exercise intolerance and increase cardiac decompensation and overall mortality in patients with HFpEF. Diuretics are established drugs to treat fluid overload and considered a cornerstone in the symptomatic therapy of HFpEF.

As small non-coding ribonucleic acid (RNA) molecules, micro-RNAs (miRNAs), such as miR-23, miR-24, miR-125, miR-195, miR-199, and miR-214, are observed to be increased in the heart tissue of patients with HF [159]. Hypertrophic growth was caused by overexpression of these miRNAs in cultured myocytes. There are different profiles of miRNAs between patients with HFpEF and HFrEF, and targeting miRNAs may initiate new treatment methods of HFpEF in the future [160]. However, mechanisms and application of treatment methods targeting miRNAs need to be better understood through further studies. Cell therapy targets myocardial inflammation and fibrosis in HFpEF and may be a promising treatment for HFpEF. It is still unclear which is the optimal cell type, dose, and delivery route in HFpEF [161].

CardioMEMS device, a radio frequency-based wireless pressure sensor, improves cardiac outcome through continuously monitoring PAP in patients with HF [162, 163]. LV mechanical dyssynchrony causes impaired LV function, higher LV-filling pressure, and worsened clinical symptoms in patients with HFpEF [164–166]. However, it may not be associated with cardiac outcomes of patients with HFpEF [167]. Further studies will assess the effects of both cardiac resynchronization therapy and cardiac contractility modulation on exercise tolerance in patients with HFpEF [168]. Renal sympathetic denervation cannot affect exercise tolerance in patients with HFpEF, although it lowers blood pressure, reduces LV mass, and improves diastolic function [169–172].

## Conclusion

With its increasing prevalence and worsening prognosis, HFpEF is nearly unique to the elderly and considered a true geriatric syndrome. Compared with the tremendous progress in the diagnosis and treatment of HFrEF, HFpEF continues to be a great enigma and needs to be further studied considering the failure of HF drugs to improve its outcome [173]. There is a lack of precise indicators for diagnosing HFpEF and a high prevalence of comorbidities that may interfere with HFpEF diagnosis [174]. Clinical trials generally enroll all participants with HF symptoms and preserved LVEF [175]. However, HFpEF is a heterogeneous syndrome with multiple phenotypes, affected by aging, and involving many organs [176]. HFpEF represents multifactorial and multisystemic syndrome with different pathophysiologies and phenotypes. Treatment with a single target fails to significantly affect HFpEF outcomes; however, lifestyle modifications prove to be an effective way to approach HFpEF as a clinical syndrome. Drugs and interventions applied to treat HFpEF have been principally based on central hemodynamic and neurohormonal abnormalities, which appear to be less complete in HFpEF than in HFrEF [177]. Individually tailored approaches may promote effective identification of HFpEF through underlying age-related changes and various comorbidities. Further extensive studies aimed to investigate HFpEF, aging, and comorbidities in carefully phenotyped HFpEF subgroups may elucidate the biology, diagnosis, and treatment of HFpEF.

## Data Availability

Not applicable.

## References

[1] Ponikowski P (2016). 2016 ESC Guidelines for the diagnosis and treatment of acute and chronic heart failure: The Task Force for the diagnosis and treatment of acute and chronic heart failure of the European Society of Cardiology (ESC) Developed with the special contribution of the Heart Failure Association (HFA) of the ESC. Eur Heart J.

[2] Paulus W, Tschope C (2013). A novel paradigm for heart failure with preserved ejection fraction: comorbidities drive myocardial dysfunction and Remodeling through coronary microvascular endothelial inflammation. J Am Coll Cardiol.

[3] Fonarow GC (2007). Characteristics, treatments, and outcomes of patients with preserved systolic function hospitalized for heart failure: a report from the OPTIMIZE-HF Registry. J Am Coll Cardiol.

[4] Kitzman DW, Shah SJ (2016). The HFpEF obesity phenotype: the elephant in the room. J Am Coll Cardiol.

[5] Solomon SD (2019). PARAGON-HF Investigators and Committees. Angiotensin-neprilysin inhibition in heart failure with preserved ejection fraction. N Engl J Med.

[6] Figtree GA (2019). Effects of canagliflozin on heart failure outcomes associated with preserved and reduced ejection fraction in type 2 diabetes: results from the CANVAS program. Circulation.

[7] van Woerden G, Gorter TM, Westenbrink BD, Willems TP, van Veldhuisen DJ, Rienstra M (2018). Epicardial fat in heart failure patients with mid-range and preserved ejection fraction. Eur J Heart Fail.

[8] Mahajan R (2018). Electroanatomical remodeling of the atria in obesity: impact of adjacent epicardial fat. JACC Clin Electrophysiol.

[9] Shah SJ (2018). Prevalence and correlates of coronary microvascular dysfunction in heart failure with preserved ejection fraction: PROMIS-HFpEF. Eur Heart J.

[10] Kanagala P (2018). Relationship between focal and diffuse fibrosis assessed by CMR and clinical outcomes in heart failure with preserved ejection fraction. JACC Cardiovasc Imaging.

[11] Siebermair J (2019). Atrial fibrosis in non-atrial fibrillation individuals and prediction of atrial fibrillation by use of late gadolinium enhancement magnetic resonance imaging. J Cardiovasc Electrophysiol.

[12] Shah SJ (2016). Phenotype-specific treatment of heart failure with preserved ejection fraction: a multiorgan roadmap. Circulation.

[13] Triposkiadis F (2019). The continuous heart failure spectrum: moving beyond an ejection fraction classifcation. Eur Heart J.

[14] Fu S, Xie L, Li D, Ye P, Luo L (2015). The predictive capacity and additional prognostic power of N-terminal pro-B-type natriuretic peptide in Chinese elderly with chronic heart failure. Clin Interv Aging.

[15] Borlaug BA (2014). The pathophysiology of heart failure with preserved ejection fraction. Nat Rev Cardiol.

[16] Kaye DM, Marwick TH (2017). Impaired right heart and pulmonary vascular function in HFpEF: time for more risk markers?. JACC Cardiovasc Imaging.

[17] Iyngkaran P, Anavekar NS, Neil C, Thomas L, Hare DL (2017). Shortness of breath in clinical practice: a case for left atrial function and exercise stress testing for a comprehensive diastolic heart failure workup. World J Methodol.

[18] Lam CSP, Donal E, Kraigher-Krainer E, Vasan RS (2011). Epidemiology and clinical course of heart failure with preserved ejection fraction. Eur J Heart Fail.

[19] Frantz S (2018). The innate immune system in chronic cardiomyopathy: a European Society of Cardiology (ESC) scientifc statement from the Working Group on Myocardial Function of the ESC. Eur J Heart Fail.

[20] Mohammed SF, Hussain S, Mirzoyev SA, Edwards WD, Maleszewski JJ, Redfield MM (2015). Coronary microvascular rarefaction and myocardial fibrosis in heart failure with preserved ejection fraction. Circulation.

[21] Gevaert AB, Boen JRA, Segers VF, Van Craenenbroeck EM (2019). Heart failure with preserved ejection fraction: a review of cardiac and noncardiac pathophysiology. Front Physiol.

[22] Mohammed SF (2014). Right ventricular function in heart failure with preserved ejection fraction: a community-based study. Circulation.

[23] Zile MR (2015). Myocardial stifness in patients with heart failure and a preserved ejection fraction: contributions of collagen and titin. Circulation.

[24] Krüger M (2009). Protein kinase G modulates human myocardial passive stiffness by phosphorylation of the titin springs. Circ Res.

[25] van Heerebeek L, Franssen CPM, Hamdani N, Verheugt FWA, Somsen GA, Paulus WJ (2012). Molecular and cellular basis for diastolic dysfunction. Curr Heart Fail Rep.

[26] Packer M (2018). Epicardial adipose tissue may mediate deleterious effects of obesity and inflammation on the myocardium. J Am Coll Cardiol.

[27] Packer M (2018). The epicardial adipose inflammatory triad: coronary atherosclerosis, atrial fibrillation, and heart failure with a preserved ejection fraction. Eur J Heart Fail.

[28] Greulich S (2012). Secretory products from epicardial adipose tissue of patients with type 2 diabetes mellitus induce cardiomyocyte dysfunction. Circulation.

[29] Fu S (2013). Overall and abdominal obesity indicators had different association with central arterial stiffness and hemodynamics independent of age, sex, blood pressure, glucose, and lipids in Chinese community-dwelling adults. Clin Interv Aging.

[30] Mazurek T (2014). Relation of proinflammatory activity of epicardial adipose tissue to the occurrence of atrial fibrillation. Am J Cardiol.

[31] Creemers EE, Pinto YM (2011). Molecular mechanisms that control interstitial fbrosis in the pressure-overloaded heart. Cardiovasc Res.

[32] de Boer RA (2019). Towards better defnition, quantifcation and treatment of fbrosis in heart failure. A scientific roadmap by the Committee of Translational Research of the Heart Failure Association (HFA) of the European Society of Cardiology. Eur J Heart Fail.

[33] Lam CSP, Voors AA, de Boer RA, Solomon SD, van Veldhuisen DJ (2018). Heart failure with preserved ejection fraction: from mechanisms to therapies. Eur Heart J.

[34] Rich MW, Kitzman DW (2000). Heart failure in octogenarians: a fundamentally different disease. Am J Geriatr Cardiol.

[35] Upadhya B, Haykowsky MJ, Eggebeen J, Kitzman DW (2015). Sarcopenic obesity and the pathogenesis of exercise intolerance in heart failure with preserved ejection fraction. Curr Heart Fail Rep.

[36] Ather S (2012). Impact of noncardiac comorbidities on morbidity and mortality in a predominantly male population with heart failure and preserved versus reduced ejection fraction. J Am Coll Cardiol.

[37] Murad K, Kitzman D (2011). Frailty and multiple comorbidities in the elderly patient with heart failure: implications for management. Heart Fail Rev.

[38] Franssen C (2015). Myocardial microvascular inflammatory endothelial activation in heart failure with preserved ejection fraction. JACC Heart Fail.

[39] Pedone C, Roshanravan B, Scarlata S, Patel KV, Ferrucci L, Incalzi RA (2015). Longitudinal association between serum leptin concentration and glomerular filtration rate in humans. PLoS ONE.

[40] Bouthoorn S (2018). The prevalence of left ventricular diastolic dysfunction and heart failure with preserved ejection fraction in men and women with type 2 diabetes: a systematic review and meta-analysis. Diab Vasc Dis Res.

[41] Schelbert EB (2017). Temporal relation between myocardial fibrosis and heart failure with preserved ejection fraction: association with baseline disease severity and subsequent outcome. JAMA Cardiol.

[42] Cheng RK (2014). Outcomes in patients with heart failure with preserved, borderline, and reduced ejection fraction in the Medicare population. Am Heart J.

[43] Packer M (2018). Do most obese people with exercise intolerance and a normal ejection fraction have treatable heart failure?. Am J Med.

[44] Packer M (2019). The conundrum of patients with obesity, exercise intolerance, elevated ventricular filling pressures and a measured ejection fraction in the normal range. Eur J Heart Fail.

[45] Fu S, Jiao J, Guo Y, Zhu B, Luo L (2019). N-terminal pro-brain natriuretic peptide levels had an independent and added ability in the evaluation of all-cause mortality in older Chinese patients with atrial fibrillation. BMC Geriatr.

[46] Sramko M (2019). Independent effect of atrial fibrillation on natriuretic peptide release. Clin Res Cardiol.

[47] Gruden G, Landi A, Bruno G (2014). Natriuretic peptides, heart, and adipose tissue: new findings and future developments for diabetes research. Diabetes Care.

[48] Yancy CW (2017). 2017 ACC/AHA/HFSA focused update of the 2013 ACCF/AHA guideline for the Management of Heart Failure. J Am Coll Cardiol.

[49] Gupta DK (2016). Effective anticoagulation with factor Xa next generation in AF–Thrombolysis in Myocardial Infarction 48 (ENGAGE AF–IMI 48) Echocardiographic Study Investigators. The prognostic significance of cardiac structure and function in atrial fibrillation: the ENGAGE AF-TIMI 48 echocardiographic substudy. J Am Soc Echocardiogr.

[50] Obakata M, Kane GC, Reddy YNV, Olson TP, Melenovsky V, Borlaug BA (2017). Role of diastolic stress testing in the evaluation for heart failure with preserved ejection fraction a simultaneous invasive-echocardiographic study. Circulation.

[51] Nagueh SF (2016). Recommendations for the evaluation of left ventricular diastolic function by echocardiography: an update from the American Society of Echocardiography and the European Association of Cardiovascular Imaging. J Am Soc Echocardiogr.

[52] Dryer K (2018). Coronary microvascular dysfunction in patients with heart failure with preserved ejection fraction. Am J Physiol Heart Circ Physiol.

[53] Yancy CW (2016). 2016 ACC/AHA/HFSA focused update on new pharmacological therapy for heart failure: an update of the 2013 ACCF/AHA guideline for the management of heart failure: a report of the American College of Cardiology/American Heart Association Task Force on Clinical Practice Guidelines and the Heart Failure Society of America. Circulation.

[54] Taqueti VR (2018). Coronary microvascular dysfunction and future risk of heart failure with preserved ejection fraction. Eur Heart J.

[55] Hussain N (2016). Pulmonary hypertension in patients with heart failure and preserved ejection fraction: differential diagnosis and management. Pulm Circ.

[56] Shah AM, Solomon SD (2012). Phenotypic and pathophysiological heterogeneity in heart failure with preserved ejection fraction. Eur Heart J.

[57] Pieske B (2019). How to diagnose heart failure with preserved ejection fraction: the HFAPEFF diagnostic algorithm: a consensus recommendation from the Heart Failure Association (HFA) of the European Society of Cardiology (ESC). Eur Heart J.

[58] Haykowsky M, Kouba EJ, Brubaker PH, Nicklas BJ, Eggebeen J, Kitzman DW (2014). Skeletal muscle composition and its relation to exercise intolerance in older patients with heart failure and preserved ejection fraction. Am J Cardiol.

[59] Kitzman DW (2016). Effect of caloric restriction or aerobic exercise training on peak oxygen consumption and quality of life in obese older patients with heart failure with preserved ejection fraction: a randomised clinical trial. JAMA.

[60] Haass M (2011). Body mass index and adverse cardiovascular outcomes in heart failure patients with preserved ejection fraction/clinical perspective. Circ Heart Fail.

[61] Rodriguez Flores M, Aguilar Salinas C, Piché ME, Auclair A, Poirier P (2017). Effect of bariatric surgery on heart failure. Expert Rev Cardiovasc Ther.

[62] van Eyk HJ (2018). Caloric restriction lowers endocannabinoid tonus and improves cardiac function in type 2 diabetes. Nutr Diabetes.

[63] Pathak RK (2015). Long-term effect of goal-directed weight management in an atrial fibrillation cohort: a long-term follow-up study (LEGACY). J Am Coll Cardiol.

[64] Sundström J, Bruze G, Ottosson J, Marcus C, Näslund I, Neovius M (2017). Weight loss and heart failure: a nationwide study of gastric bypass surgery versus intensive lifestyle treatment. Circulation.

[65] Reeves GR (2016). A novel rehabilitation intervention for older patients with acute decompensated heart failure: the REHAB-HF pilot study. JACC Heart Fail.

[66] Dunlay SM, Roger VL, Redfeld MM (2017). Epidemiology of heart failure with preserved ejection fraction. Nat Rev Cardiol.

[67] Tschöpe C (2018). Heart failure with preserved ejection fraction: current management and future strategies: expert opinion on the behalf of the Nucleus of the “Heart Failure Working Group” of the German Society of Cardiology (DKG). Clin Res Cardiol.

[68] Williams B (2018). 2018 ESC/ESH Guidelines for the management of arterial hypertension. Eur Heart J.

[69] Williamson JD (2016). Intensive vs standard blood pressure control and cardiovascular disease outcomes in adults aged >/=75 years: a randomized clinical trial. JAMA.

[70] Yancy CW (2013). 2013 ACCF/AHA guideline for the management of heart-failure: a report of the American College of Cardiology Foundation/American Heart Association task force on practice guidelines. J Am Coll Cardiol.

[71] Gandhi SK (2001). The pathogenesis of acute pulmonary edema associated with hypertension. N Engl J Med.

[72] Marrouche NF (2018). Catheter ablation for atrial fbrillation with heart failure. N Engl J Med.

[73] Lam CS (2016). Atrial fibrillation in heart failure with preserved ejection fraction: association with exercise capacity, left ventricular filling pressures, natriuretic peptides, and left atrial volume. JACC Heart Fail.

[74] Eitel C (2019). Atrial fbrillation ablation strategies and outcome in patients with heart failure: insights from the German ablation registry. Clin Res Cardiol.

[75] Machino-Ohtsuka T (2013). Efficacy, safety, and outcomes of catheter ablation of atrial fibrillation in patients with heart failure with preserved ejection fraction. J Am Coll Cardiol.

[76] Dunlay SM, Weston SA, Redfield MM, Killian JM, Roger VL (2008). Anemia and heart failure: a community study. Am J Med.

[77] Anker SD (2018). Effects of ferric carboxymaltose on hospitalisations and mortality rates in iron-defcient heart failure patients: an individual patient data meta-analysis. Eur J Heart Fail.

[78] Stugiewicz M, Tkaczyszyn M, Kasztura M, Banasiak W, Ponikowski P, Jankowska EA (2016). The infuence of iron defciency on the functioning of skeletal muscles: experimental evidence and clinical implications. Eur J Heart Fail.

[79] Kaschina E, Unger T (2003). Angiotensin AT1/AT2 receptors: regulation, signalling and function. Blood Press.

[80] Pahlavani M, Kalupahana NS, Ramalingam L, Moustaid-Moussa N (2017). Regulation and functions of the renin–angiotensin system in white and brown adipose tissue. Compr Physiol.

[81] Ghanim H, Monte S, Caruana J, Green K, Abuaysheh S, Dandona P (2018). Decreases in neprilysin and vasoconstrictors and increases in vasodilators following bariatric surgery. Diabetes Obes Metab.

[82] Wang Q (2017). The crucial role of activin A/ALK4 pathway in the pathogenesis of Ang-II-induced atrial fibrosis and vulnerability to atrial fibrillation. Basic Res Cardiol.

[83] Goossens GH (2012). Valsartan improves adipose tissue function in humans with impaired glucose metabolism: a randomized placebo-controlled double-blind trial. PLoS ONE.

[84] Massie BM (2008). Irbesartan in patients with heart failure and preserved ejection fraction. N Engl J Med.

[85] Yip GWK (2008). The Hong Kong diastolic heart failure study: a randomised controlled trial of diuretics, irbesartan and ramipril on quality of life, exercise capacity, left ventricular global and regional function in heart failure with a normal ejection fraction. Heart.

[86] Seferovic PM (2019). Clinical practice update on heart failure 2019: pharmacotherapy, procedures, devices and patient management. An expert consensus meeting report of the Heart Failure Association of the European Society of Cardiology. Eur J Heart Fail.

[87] Brown NJ (2005). Aldosterone and end-organ damage. Curr Opin Nephrol Hypertens.

[88] Pitt B (2014). Spironolactone for heart failure with preserved ejection fraction. N Engl J Med.

[89] Hunt SA (2009). 2009 focused update incorporated into the ACC/AHA 2005 Guidelines for the Diagnosis and Management of Heart Failure in Adults: a report of the American College of Cardiology Foundation/American Heart Association Task Force on Practice Guidelines: developed in collaboration with the International Society for Heart and Lung Transplantation. Circulation.

[90] Fukuta H, Goto T, Wakami K, Ohte N (2017). The effect of betablockers on mortality in heart failure with preserved ejection fraction: a meta-analysis of observational cohort and randomized controlled studies. Int J Cardiol.

[91] Belge C (2014). Enhanced expression of beta3-adrenoceptors in cardiac myocytes attenuates neurohormone-induced hypertrophic remodeling through nitric oxide synthase. Circulation.

[92] Pal N (2015). Effect of selective heart rate slowing in heart failure with preserved ejection fraction. Circulation.

[93] Domínguez E (2018). Heart rate response and functional capacity in patients with chronic heart failure with preserved ejection fraction. ESC Heart Fail.

[94] Kosmala W, Holland DJ, Rojek A, Wright L, Przewlocka-Kosmala M, Marwick TH (2013). Effect of If-channel inhibition on hemodynamic status and exercise tolerance in heart failure with preserved ejection fraction: a randomized trial. J Am Coll Cardiol.

[95] Sartipy U, Savarese G, Dahlström U, Fu M, Lund LH (2019). Association of heart rate with mortality in sinus rhythm and atrial fibrillation in heart failure with preserved ejection fraction. Eur J Heart Fail.

[96] Whitbeck MG (2013). Increased mortality among patients taking digoxin-analysis from the AFFIRM study. Eur Heart J.

[97] Khazanie P, Hellkamp AS, Fonarow GC, Curtis LH, Al-Khatib SM, Hernandez AF (2018). Permanent pacemaker use among patients with heart failure and preserved ejection fraction: findings from the Acute Decompensated Heart Failure National Registry (ADHERE) National Registry. Am Heart J.

[98] Voskoboinik A (2019). Cardioversion of atrial fibrillation in obese patients: results from the Cardioversion-BMI randomized controlled trial. J Cardiovasc Electrophysiol.

[99] Mareev Y, Cleland JG (2015). Should B-blockers be used in patients with heart failure and atrial fibrillation?. Clin Ther.

[100] Eschalier R (2014). Features of cardiac remodeling, associated with blood pressure and fibrosis biomarkers, are frequent in subjects with abdominal obesity. Hypertension.

[101] Thomas L, Marwick TH, Popescu BA, Donal E, Badano LP (2019). Left atrial structure and function, and left ventricular diastolic dysfunction: JACC state-of-the-art review. J Am Coll Cardiol.

[102] Hirai T, Cotseones G, Makki N, Agrawal A, Wilber DJ, Barron JT (2014). Usefulness of left ventricular diastolic function to predict recurrence of atrial fibrillation in patients with preserved left ventricular systolic function. Am J Cardiol.

[103] Ling LH (2013). Sinus rhythm restores ventricular function in patients with cardiomyopathy and no late gadolinium enhancement on cardiac magnetic resonance imaging who undergo catheter ablation for atrial fibrillation. Heart Rhythm.

[104] Fukumoto K (2015). Comparison of preexisting and ablation-induced late gadolinium enhancement on left atrial magnetic resonance imaging. Heart Rhythm.

[105] Packer M (2019). Effect of catheter ablation on pre-existing abnormalities of left atrial systolic, diastolic, and neurohormonal functions in patients with chronic heart failure and atrial fibrillation. Eur Heart J.

[106] Urey MA (2017). Stiff left atrial syndrome after multiple percutaneous catheter ablations: role for invasive hemodynamic exercise testing. Circ Heart Fail.

[107] Parisi V (2019). Statin therapy modulates thickness and inflammatory profile of human epicardial adipose tissue. Int J Cardiol.

[108] Yang Q, Qi X, Dang Y, Li Y, Song X, Hao X (2016). Effects of atorvastatin on atrial remodeling in a rabbit model of atrial fibrillation produced by rapid atrial pacing. BMC Cardiovasc Disord.

[109] Flint AC (2017). Statin adherence is associated with reduced recurrent stroke risk in patients with or without atrial fibrillation. Stroke.

[110] Preiss D (2015). The effect of statin therapy on heart failure events: a collaborative meta-analysis of unpublished data from major randomized trials. Eur Heart J.

[111] Fu S, Ping P, Ye P, Luo L (2019). Relationship between drug application and mortality rate in Chinese older coronary artery disease/chronic heart failure patients with and without low glomerular filtration rate. BMC Pharmacol Toxicol.

[112] Karayannis G (2013). Association between epicardial fat thickness and weight homeostasis hormones in patients with noncachectic heart failure. Angiology.

[113] Solomon SD (2012). Prospective comparison of ARNI with ARB on Management Of heart failUre with preserved ejectioN fracTion (PARAMOUNT) Investigators. The angiotensin receptor neprilysin inhibitor LCZ696 in heart failure with preserved ejection fraction: a phase 2 double-blind randomised controlled trial. Lancet.

[114] Fu S, Ping P, Wang F, Luo L (2018). Synthesis, secretion, function, metabolism and application of natriuretic peptides in heart failure. J Biol Eng.

[115] Fu S, Ping P, Zhu Q, Ye P, Luo L (2018). Brain natriuretic peptide and its biochemical, analytical, and clinical issues in heart failure: a narrative review. Front Physiol.

[116] Yusuf S (2003). Effects of candesartan in patients with chronic heart failure and preserved left-ventricular ejection fraction: the CHARM-preserved trial. Lancet.

[117] Tschöpe C, Van Linthout S (2014). New insights in (inter) cellular mechanisms by heart failure with preserved ejection fraction. Curr Heart Fail Rep.

[118] Redfeld MM (2015). Isosorbide mononitrate in heart failure with preserved ejection fraction. N Engl J Med.

[119] Borlaug BA (2018). Effect of inorganic nitrite vs placebo on exercise capacity among patients with heart failure with preserved ejection fraction: the INDIE-HFpEF randomized clinical trial. JAMA.

[120] Westermann D (2009). Enhancement of the endothelial NO synthase attenuates experimental diastolic heart failure. Basic Res Cardiol.

[121] Schiattarella GG (2019). Nitrosative stress drives heart failure with preserved ejection fraction. Nature.

[122] Redfeld MM (2013). Efect of phosphodiesterase-5 inhibition on exercise capacity and clinical status in heart failure with preserved ejection fraction: a randomized clinical trial. JAMA.

[123] Hoendermis ES (2015). Efects of sildenafl on invasive haemodynamics and exercise capacity in heart failure patients with preserved ejection fraction and pulmonary hypertension: a randomized controlled trial. Eur Heart J.

[124] Guazzi M, Vicenzi M, Arena R, Guazzi MD (2011). PDE5 inhibition with sildenafl improves left ventricular diastolic function, cardiac geometry, and clinical status in patients with stable systolic heart failure: results of a 1-year, prospective, randomized, placebo-controlled study. Circ Heart Fail.

[125] Filippatos G (2017). Patient-reported outcomes in the SOluble guanylate Cyclase stimulatoR in heArT failurE patientS with PRESERVED ejection fraction (SOCRATES-PRESERVED) study. Eur J Heart Fail.

[126] Cieslik KA, Trial J, Carlson S, Taffet GE, Entman ML (2013). Aberrant differentiation of fibroblast progenitors contributes to fibrosis in the aged murine heart: role of elevated circulating insulin levels. FASEB J.

[127] Bell DSH, Goncalves E (2019). Atrial fibrillation and type 2 diabetes: prevalence, etiology, pathophysiology and effect of anti-diabetic therapies. Diabetes Obes Metab.

[128] Liou YS, Yang FY, Chen HY, Jong GP (2018). Antihyperglycemic drugs use and new-onset atrial fibrillation: a population-based nested case control study. PLoS ONE.

[129] Jing Y, Wu F, Li D, Yang L, Li Q, Li R (2018). Metformin improves obesity-associated inflammation by altering macrophages polarization. Mol Cell Endocrinol.

[130] Chang SH (2014). Association of metformin with lower atrial fibrillation risk among patients with type 2 diabetes mellitus: a population-based dynamic cohort and in vitro studies. Cardiovasc Diabetol.

[131] Yang W, Yang C, Luo J, Wei Y, Wang W, Zhong Y (2018). Adiponectin promotes preadipocyte differentiation via the PPARpathway. Mol Med Rep.

[132] Antonopoulos AS (2016). Mutual regulation of epicardial adipose tissue and myocardial redox state by PPAR-/adiponectin signalling. Circ Res.

[133] Pallisgaard JL (2017). Thiazolidinediones are associated with a decreased risk of atrial fibrillation compared with other antidiabetic treatment: a nationwide cohort study. Eur Heart J Cardiovasc Pharmacother.

[134] Clarke GD (2017). Pioglitazone improves left ventricular diastolic function in subjects with diabetes. Diabetes Care.

[135] Zhang Z (2017). Thiazolidinedione use and atrial fibrillation in diabetic patients: a meta-analysis. BMC Cardiovasc Disord.

[136] Hernandez AV, Usmani A, Rajamanickam A, Moheet A (2011). Thiazolidine-diones and risk of heart failure in patients with or at high risk of type 2 diabetes mellitus: a meta-analysis and meta-regression analysis of placebo-controlled randomized clinical trials. Am J Cardiovasc Drugs.

[137] Cacciapuoti F, Magro VM, Caturano M, Lama D, Cacciapuoti F (2017). The role of ivabradine in diastolic heart failure with preserved ejection fraction. A Doppler-echocardiographic study. J Cardiovasc Echogr.

[138] McMurray JJV (2019). Dapaglifozin in patients with heart failure and reduced ejection fraction. N Engl J Med.

[139] Maack C (2018). Heart failure and diabetes: metabolic alterations and therapeutic interventions: a state-of-the-art review from the Translational Research Committee of the Heart Failure Association-European Society of Cardiology. Eur Heart J.

[140] Verma S, McMurray JJV, Cherney DZI (2017). The metabolodiuretic promise of sodium-dependent glucose cotransporter 2 inhibition: the search for the sweet spot in heart failure. JAMA Cardiol.

[141] Pabel S (2018). Empaglifozin directly improves diastolic function in human heart failure. Eur J Heart Fail.

[142] Yagi S (2017). Canagliflozin reduces epicardial fat in patients with type 2 diabetes mellitus. Diabetol Metab Syndr.

[143] Inzucchi SE, McGuire DK (2008). New drugs for the treatment of diabetes: part II: incretin-based therapy and beyond. Circulation.

[144] Marso SP (2016). Semaglutide and cardiovascular outcomes in patients with type 2 diabetes. N Engl J Med.

[145] Graziani F, Varone F, Crea F, Richeldi L (2018). Treating heart failure with preserved ejection fraction: learning from pulmonary fbrosis. Eur J Heart Fail.

[146] Yamagami K (2015). Pirfenidone exhibits cardioprotective efects by regulating myocardial fbrosis and vascular permeability in pressure-overloaded hearts. Am J Physiol Heart Circ Physiol.

[147] Hartog JWL, Voors AA, Bakker SJL, Smit AJ, van Veldhuisen DJ (2007). Advanced glycation end-products (AGEs) and heart failure: pathophysiology and clinical implications. Eur J Heart Fail.

[148] Kasner M (2011). Diastolic tissue Doppler indexes correlate with the degree of collagen expression and cross-linking in heart failure and normal ejection fraction. J Am Coll Cardiol.

[149] Little WC, Zile MR, Kitzman DW, Hundley WG, O’Brien TX, Degroof RC (2005). The effect of alagebrium chloride (ALT-711), a novel glucose cross-link breaker, in the treatment of elderly patients with diastolic heart failure. J Card Fail.

[150] Yang J (2016). Targeting LOXL2 for cardiac interstitial fibrosis and heart failure treatment. Nat Commun.

[151] Van Tassell BW (2018). IL-1 blockade in patients with heart failure with preserved ejection fraction. Circ Heart Fail.

[152] Everett BM (2019). Anti-infammatory therapy with canakinumab for the prevention of hospitalization for heart failure. Circulation.

[153] Bertero E, Maack C (2018). Calcium signaling and reactive oxygen species in mitochondria. Circ Res.

[154] Sabbah HN, Gupta RC, Kohli S, Wang M, Hachem S, Zhang K (2016). Chronic therapy with elamipretide (MTP-131), a novel mitochondria-targeting peptide, improves left ventricular and mitochondrial function in dogs with advanced heart failure. Circ Heart Fail.

[155] Daubert MA (2017). Novel mitochondria-targeting peptide in heart failure treatment: a randomized, placebo-controlled trial of elamipretide. Circ Heart Fail.

[156] Voors AA (2018). Rationale and design of the phase 2b clinical trials to study the efects of the partial adenosine A1-receptor agonist neladenoson bialanate in patients with chronic heart failure with reduced (PANTHEON) and preserved (PANACHE) ejection fraction. Eur J Heart Fail.

[157] Parissis JT (2006). Effects of levosimendan on right ventricular function in patients with advanced heart failure. Am J Cardiol.

[158] Grossman NL, Fiack CA, Weinberg JM, Rybin DV, Farber HW (2015). Pulmonary hypertension associated with heart failure with preserved ejection fraction: acute hemodynamic effects of inhaled iloprost. Pulm Circ.

[159] Kukreja RC, Yin C, Salloum FN (2011). MicroRNAs: new players in cardiac injury and protection. Mol Pharmacol.

[160] Wong LL (2015). Circulating microRNAs in heart failure with reduced and preserved left ventricular ejection fraction. Eur J Heart Fail.

[161] Psaltis PJ, Schwarz N, Toledo-Flores D, Nicholls SJ (2016). Cellular therapy for heart failure. Curr Cardiol Rev.

[162] Adamson PB (2011). CHAMPION trial rationale and design: the long-term safety and clinical efcacy of a wireless pulmonary artery pressure monitoring system. J Card Fail.

[163] Verdejo HE (2007). Comparison of a radiofrequency-based wireless pressure sensor to swan-ganz catheter and echocardiography for ambulatory assessment of pulmonary artery pressure in heart failure. J Am Coll Cardiol.

[164] Lutembacher R (1916). De la tenose mitrale avec communication interauirulaier. Arch Mal Coeur Vaiss.

[165] Hasenfuss G (2016). A transcatheter intracar diac shunt device for heart failure with preserved ejection fraction (REDUCE LAP-HF): a multicentre, open-label, single-arm, phase 1 trial. Lancet.

[166] Morris DA, Vaz Perez A, Blaschke F, Eichstadt H, Ozcelik C, Haverkamp W (2012). Myocardial systolic and diastolic consequences of left ventricular mechanical dyssynchrony in heart failure with normal left ventricular ejection fraction. Eur Heart J Cardiovasc Imaging.

[167] Biering-Sorensen T (2017). Prognostic importance of left ventricular mechanical dyssynchrony in heart failure with preserved ejection fraction. Eur J Heart Fail.

[168] Tschöpe C (2019). Cardiac contractility modulation: mechanisms of action in heart failure with reduced ejection fraction and beyond. Eur J Heart Fail.

[169] Krum H (2009). Catheter-based renal sympathetic denervation for resistant hypertension: a multicentre safety and proof of-principle cohort study. Lancet.

[170] Donazzan L (2016). Effects of catheter-based renal denervation on cardiac sympathetic activity and innervation in patients with resistant hypertension. Clin Res Cardiol.

[171] Schirmer SH (2015). Atrial remodeling following catheter-based renal denervation occurs in a blood pressure and heart rate-independent manner. JACC Cardiovasc Interv.

[172] Patel HC (2016). Renal denervation in heart failure with preserved ejection fraction (RDT-PEF): a randomized controlled trial. Eur J Heart Fail.

[173] Wintrich J (2020). Therapeutic approaches in heart failure with preserved ejection fraction: past, present, and future. Clin Res Cardiol.

[174] Upadhya B, Kitzman DW (2020). Heart failure with preserved ejection fraction: new approaches to diagnosis and management. Clin Cardiol.

[175] Iyngkaran P (2020). The heart failure with preserved ejection fraction conundrum-redefining the problem and finding common ground?. Curr Heart Fail Rep.

[176] Packer M (2020). Do most patients with obesity or type 2 diabetes, and atrial fibrillation, also have undiagnosed heart failure? A critical conceptual framework for understanding mechanisms and improving diagnosis and treatment. Eur J Heart Fail.

[177] Borlaug BA (2014). Exercise haemodynamics and outcome in patients with dyspnoea. Eur Heart J.

